# O'nyong-nyong Virus, Chad

**DOI:** 10.3201/eid1208.060199

**Published:** 2006-08

**Authors:** Maël Bessaud, Christophe N. Peyrefitte, Boris A.M. Pastorino, Patrick Gravier, Fabienne Tock, Fabrice Boete, Hugues J. Tolou, Marc Grandadam

**Affiliations:** * Institut de médecine tropicale du Service de santé des armées, Marseille, France;; † Cabinet médical d'unité, Commercy, France

**Keywords:** O’nyong-nyong virus, Chad, Alphavirus, dispatch

## Abstract

We report the first laboratory-confirmed human infection with O'nyong-nyong virus in Chad. This virus was isolated from peripheral blood mononuclear cells of a patient with evidence of a seroconversion to a virus related to Chikungunya virus. Genome sequence was partly determined, and phylogenetic studies were conducted.

On November 2, 2004, a febrile 19-year-old French soldier staying in Chad and returning from a mission in Sarh, in the southern part of the country, was admitted to the hospital. Clinical examination showed a high body temperature (38°C), rash, periocular erythema, and pharyngitis. Abdominal, cardiopulmonary, and neurologic functions were normal. Except for body temperature, biochemical and hematologic values were normal. Serologic results were negative for *Rickettsia typhi*, *R. conorii*, *Legionella pneumophila*, *Bordetella pertussis*, HIV, and human herpesviruses 1 and 2. Results of malaria testing were also negative. The patient received intravenous acetaminophen for 2 days, according to the protocol used by the French Armed Forces Medical Service in the event of fever occurring overseas. He recovered after 5 days without sequelae.

Serum samples, collected during the acute phase (November 3–5, 2004) and after (November 23 and December 7, 2004; January 10 and February 1, 2005) were transported to our laboratory and tested by ELISA for immunoglobulin M (IgM) and IgG antibodies to a battery of arboviruses by IgM-antibody capture (MAC-ELISA) and antigen-capture ELISA, respectively (*1*). Each serum sample was considered positive if the optical density (OD) ratio, OD (viral antigen)/OD (uninfected cells), was >3. The first sample (November 3, 2004) contained no antibodies (OD ratio <2) to dengue viruses, West Nile virus, Wesselsbron virus, Rift Valley fever virus, Bunyamwera virus, or Chikungunya virus (CHIKV). Remaining samples contained antibodies to a virus serologically related to CHIKV (OD ratios >3) for both IgM (sample 2 and following samples) and IgG (sample 3 and those following). Antibody titers peaked 20 days (IgM) and 68 days (IgG) after the onset of symptoms (Figure 1). The IgM titer returned to a low level within 2 months after onset of illness.

**Figure 1:**
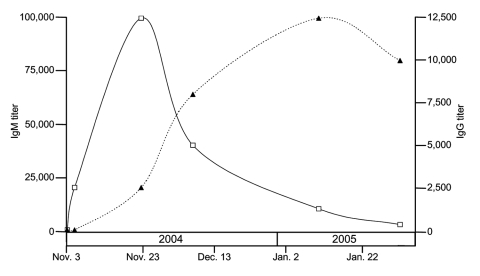
Immunoglobulin M (IgM) (□) and IgG (▲) titer in serum. For all samples, antibody titers were determined by serial dilution assays. Antibody titer is defined as the reciprocal of the highest dilution of serum that yields a positive serologic reaction.

Results of CHIKV-specific real-time reverse transcription–PCRs (*2*) performed with serum samples as templates were negative. Virus isolation was attempted by incubation of peripheral blood mononuclear cells collected on the day of onset with C6/36 (*Aedes albopictus*) and Vero (E6 clone) monolayers. After 5 days, supernatants were collected and used to infect fresh cell cultures. After 2 days, cytopathologic effects were observed in Vero monolayers; a high level of cell death was also observed in C6/36 cells. Infected Vero and C6/36 cells were then examined by indirect immunofluorescence assay (IFA) for 8 different alphaviruses with alphavirus-specific antibodies and in-house mouse hyperimmune ascitic fluids to CHIKV, Mayaro (MAYV), Tonate (TONV), Semliki Forest (SFV), and Sindbis (SINV) viruses. Results of IFA were positive when alphavirus-specific antibodies and antibodies to CHIKV, MAYV, and TONV were used; no fluorescence was observed when antibodies to SFV and SINV were used at the dilution 1:200.

CHIKV-specific real-time RT-PCRs were negative when cell culture supernatants were used as samples; this result excluded CHIKV as the etiologic agent. We next used cM3W and M2W2 alphavirus-specific primers (*3*) to partially amplify viral genome by RT-PCR. The RT-PCR product was sequenced (GenBank accession no. DQ381540, isolate IMTSSA/5163) and used in a BLAST search that identified O'nyong-nyong virus (ONNV, E value 6e^-130^).

Viral RNA was amplified for phylogenetic studies by using ONNV-specific primers for nsP3, E2, and E1 sequences (primer sequences are available on request). RT-PCR products (614, 728, and 1080-nt long, respectively) were sequenced (GenBank accession nos. DQ383272, DQ383273, and DQ399055, respectively) and compared with ONNV sequences available on GenBank database; alignments were performed with ClustalW 1.7 software. Comparison of partial sequences showed a high degree of homology between the virus we isolated in Chad and strains previously isolated. In all 4 regions sequenced, paired identity at the nucleotide and amino acid level ranged from 92% to 98% and from 95% to 98%, respectively. Compared with ONNV isolate Gulu, the nsP3 sequence of our isolate featured a glycine-encoding codon deletion (nt 5249–5251, according to ONNV strain Gulu numbering). This deletion was also observed in ONNV strains SG650 and IbH10964, which might indicate a common lineage.

Four phylograms were constructed, each based on 1 genomic region we sequenced. Among ONNV sequences, all 4 phylograms exhibited a similar pattern: ONNV isolate IbH 10964 (Nigeria) and ONNV strain SG650 (Uganda) seemed to be closely related, whereas isolates Gulu (Uganda) and IMTSSA/5163 (Chad) were placed in 2 different branches (100% bootstrap value in the 4 phylograms). The phylogenetic tree based on E1-encoding sequence (1080-nt long) gave the opportunity to include 2 other ONNV sequences (Figure 2).

**Figure 2:**
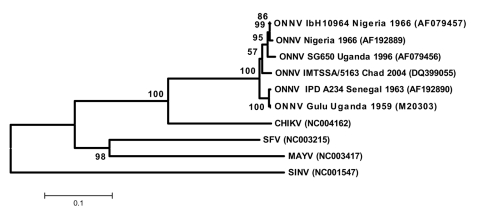
Phylogenetic tree of O'nyong-nyong virus (ONNV) based on partial E1 nucleotide sequence. Phylogram was constructed with MEGA 2 program (http://megasoftware.net/mega2.html) and tree drawing used the Juke-Cantor algorithm for genetic distance determination and the neighbor joining method. The percentage of successful bootstrap replicates (1,000 bootstrap replications, confidence probability >90%) is indicated at nodes. The length of the branches is proportional to the number of nucleotide changes (% of divergence). CHIKV (Chikungunya virus), SFV (Semliki Forest virus), MAYV (Mayaro virus), and SINV (Sindbis virus) sequences have been introduced for correct rooting of the tree.

ONNV (family *Togaviridae*, genus *Alphavirus*) was first isolated from human blood and anopheline mosquitoes in Gulu, Uganda, in 1959 (*4*) and has been responsible for several outbreaks in humans that occurred in East Africa (Kenya, Uganda, Tanzania, Malawi, Mozambique). Fever, headache, joint pains, and rash were the principal signs and symptoms (*5**,**6*). Virus isolations from human and animal sera as well as from *Anopheles funestus* and *A. gambiae* have been reported in East Africa (*7**,**8*). Human and animal infections based on serologic evidence have also been reported in Nigeria, Ghana, and Sierra Leone (*9**,**10*). ONNV was also isolated from sentinel mice in Senegal and caused an outbreak in Côte d'Ivoire in the 1980s (*11*). To our knowledge, ONNV had never before been isolated in Chad.

The Sarh region, where the patient was infected, consists predominantly of plains covered with a mixture of grasses and woodlands. This region receives heavy rainfall during the 6-month rainy season, from May to October. To our knowledge, no recent data are available concerning the presence of *Anopheles* spp. in this region. Patient infection occurred outside any reported outbreak involving ONNV or another arbovirus. Moreover, the mission involved 9 other French soldiers whose serum specimens, collected a few weeks after their return from Sarh and transported to our laboratory, did not show serologic evidence of infection with an alphavirus.

SFV antibodies failed to detect ONNV by IFA, although both viruses are members of the same antigenic complex (*12*). The distribution of ONNV strains observed in the phylograms seemed to be independent of viral isolation locations or years. This finding suggests either a high level of viral genomic sequence stability over time or the circulation of ONNV strains across Africa, which has given rise to a mixing of ONNV strains from different origins in the same areas. However, because of the limited number of sequences available for genetic comparison, this observation on the distribution of ONNV strains needs confirmation.

In the absence of virus isolation, the diagnosis of infections with ONNV is difficult because of the close antigenic relationship of this virus with other alphaviruses, especially CHIKV. Development of a specific serologic assay for ONNV within the SFV antigenic complex would be a valuable tool for diagnosis and surveillance studies.
